# Editorial: Nutrition and Health-Related Quality of Life: Is It an Ignored Outcome?

**DOI:** 10.3389/fnut.2021.778816

**Published:** 2021-10-07

**Authors:** Leila Itani, Rosa Sammarco, Marwan El Ghoch

**Affiliations:** ^1^Department of Nutrition and Dietetics, Faculty of Health Sciences, Beirut Arab University, Beirut, Lebanon; ^2^Internal Medicine and Clinical Nutrition Unit, Department of Clinical Medicine and Surgery, Federico II University Hospital, Naples, Italy

**Keywords:** HRQOL – Health-related quality of life, clinical nutrition, obesity, cardio metabolic health, mortality, Ketodiet, clinical outcomes

The concept of quality of life (QoL) represents the well-being of people living in a certain society, broadly including physical health, family, education, employment, wealth, religious beliefs, finance and the environment (1). In the last three decades, a new dimension of QoL has increased in interest and has become known as the “health-related quality of life” (HRQoL) (2), which assesses how the individual's well-being may be affected over time, either as a result of a disease, disability or disorder. In fact, the research which focused on HRQoL is extremely important, since its assessment helps monitor progress in terms of achieving the nation's health objectives (3), through its influence on current and future treatments, and health protocols across a wide spectrum of diseases (4).

On the other hand, nutrition is a vital process through which human beings retrieve energy needed for reproduction, growth and development, as well as health maintenance (5). In fact, over- and malnutrition are both associated with medical diseases (6) and psychological disorders (7). It must be remembered that the management of many of these conditions is the result of adequate nutrition (8–10). However, and despite this fact, there is a lack of knowledge relating to the link between nutrition and HRQoL (6), consequently, our Research Topic is entitled: “*Nutrition and Health-Related Quality of Life: Is it an Ignored Outcome?*” so as to attract investigators from different backgrounds, interested in both areas, namely, human nutrition and HRQoL, in order to clarify the link between the two and the nature of their interaction.

We received 17 submissions; six of these were declined following an initial editorial assessment. Eleven papers were accepted after one or more rounds of peer revision as follows: 1 clinical trial, 7 original research documents, 1 systematic review, 1 data report and 1 commentary, sourced from 11 different countries.

Di Iorio et al. reported on the beneficial effect of 30-min monthly sessions over 12 months in patients with type 2 diabetes mellitus, using an “Individualized Nutritional Therapy,” based on counting carbohydrates, which improved the patients' state of health, preventing cardiovascular risk and exponentially impacting their QoL.

Yue et al., in a pilot study, tested the feasibility and impact of a nutritional support strategy on the clinical outcomes of severe and critical patients with SARS-CoV-2 pneumonia, based on underfeeding, which restricted non-protein calories but preserved protein intake. Following the same theme, De Pipaòn et al. argued in a commentary that consumer reports of “Keto Flu” were associated with a ketogenic diet.

Liu et al. conducted a large, multicentre, prospective study, composed of 9,996 participants, aged 65 years and older. Their nutritional status and HRQoL were measured using the Mini Nutritional Assessment—Short Form (MNA-SF) and the EuroQoL, respectively. The authors found that higher MNA-SF scores were related to an improved HRQoL.

Chen et al. identified an inflammatory-nutritional marker in a study composed of patients with acute kidney injury (AKI), that could predict mortality in this population; a higher PCT to Albumin ratio was strongly associated with higher mortality in sepsis-induced AKI patients.

Gathercole et al. compared the impact of the two dietary interventions modification of dietary protein intake over a period of 10 weeks on the host fecal proteome in elderly males, who either met the minimum dietary protein recommendations (RDA) or consumed twice the recommended dietary allowance (2RDA).

Lachaud et al. examined the housing trajectories of homeless people with mental illness over a follow-up period of 6 years, and the association of these trajectories with food security. Authors in this study reported that individuals with substance use disorder, who never moved into stable accommodation, had the lowest food security status.

Wu et al. investigated the associations of diet quality, physical activity (PA), sedentary behaviors (SB) and HRQoL among children with mental health disorders. They found that health promotion programs, which focused on promoting a high-quality diet, increased PA, a better HRQoL and reduced SB among children, could contribute to improving mental health.

Gao et al. evaluated the effect of home enteral nutrition on nutritional status, body composition (BC), HRQoL and other clinical outcomes in malnourished patients with intestinal failure. It was found that home enteral nutrition improves nutritional status, BC and HRQoL.

Wang et al. conducted a systematic review of controlled trials (RCTs) to explore the efficacy of a low-FODMAP diet (LFD) with regard to alleviating the symptoms of irritable bowel syndrome (IBS). They found that an LFD is effective in reducing the global symptoms and improving the bowel habits of adult patients with IBS.

Leão et al. explored the association between nutritional status and functional status among older adults receiving assistance from the in-home nursing care service. The primary finding of this study was that better functional status is directly associated with good nutritional status.

All the studies included in this Research Topic either directly or indirectly explored the link between nutrition and HRQoL, based upon which we can identify two types of interaction: (1) bi-direction interaction: in other words, a good nutritional status leads to a better HRQoL and vice-versa or (2) synchronic interaction: the two interact with one other to impact another outcome, i.e., an adequate nutritional status plus adoption of a good QoL may improve a medical disease or a psychological disorder. Clearly, future research is still needed to replicate these findings and to consolidate them.

In conclusion, we are grateful to “Frontiers in Nutrition” for giving us the opportunity to serve as editors for this Research Topic; it has been such a challenging and motivating experience from which we have learned a great deal and which we intend to continue. Secondly, we would like to thank our valuable authors for sharing their research in this collection, which we believe will have relevance for the readership in their clinical practice. Last but not least, we wish to thank our reviewers for their time and input, which undoubtedly improved the quality of our studies.

## Author Contributions

All authors claim authorship, and have approved and made substantial contributions to the conception, drafting and final version of the paper.

## Conflict of Interest

The authors declare that the research was conducted in the absence of any commercial or financial relationships that could be construed as a potential conflict of interest.

## Publisher's Note

All claims expressed in this article are solely those of the authors and do not necessarily represent those of their affiliated organizations, or those of the publisher, the editors and the reviewers. Any product that may be evaluated in this article, or claim that may be made by its manufacturer, is not guaranteed or endorsed by the publisher.

## References

[1] Kaplan RM Ries AL. Quality of life: concept and definition. COPD. (2007) 4:263–71. 10.1080/15412550701480356 17729071

[2] Garratt A Schmidt L Mackintosh A Fitzpatrick R. Quality of life measurement: bibliographic study of patient assessed health outcome measures. BMJ. (2002) 324:1417. 10.1136/bmj.324.7351.1417 12065262 PMC115850

[3] Hesse BW Gaysynsky A Ottenbacher A Moser RP Blake KD Chou WY. Meeting the healthy people 2020 goals: using the Health Information National Trends Survey to monitor progress on health communication objectives. J Health Commun. (2014) 18:1497–509. 10.1080/10810730.2014.954084 25491584 PMC4266162

[4] Testa MA Simonson DC. Assessment of quality-of-life outcomes. N Engl J Med. (1996) 334:835–40. 10.1056/NEJM199603283341306 8596551

[5] WHO. Nutrition. WHO. Available online at:https://www.who.int/health/topics/nutrition (accessed September 10, 2021).

[6] El Ghoch M El Shamieh S. Is there a link between nutrition, genetics, and cardiovascular disease? J Cardiovasc Dev Dis. (2020) 7:33. 10.3390/jcdd7030033 32867398 PMC7570096

[7] Laessle RG Schweiger U Pirke KM. Depression as a correlate of starvation in patients with eating disorders. Biol Psychiatry. (1988) 23:19–25. 3370268 10.1016/0006-3223(88)90056-x

[8] Muscogiuri G Barrea L Caprio M Ceriani F Chavez AO El Ghoch M. Nutritional guidelines for the management of insulin resistance. Crit Rev Food Sci Nutr. (2021) 2021:1–14. 10.1080/10408398.2021.1908223 33797999

[9] El Ghoch M Gatti D Calugi S Viapiana O Bazzani PV Dalle Grave R. The association between weight gain/restoration and bone mineral density in adolescents with anorexia nervosa: a systematic review. Nutrients. (2016) 8:769. 10.3390/nu8120769 27916839 PMC5188424

[10] Chehade L Jaafar ZA El Masri D Zmerly H Kreidieh D Tannir H. Lifestyle modification in rheumatoid arthritis: dietary and physical activity recommendations based on evidence. Curr Rheumatol Rev. (2019) 15:209–14. 10.2174/1573397115666190121135940 30666911

